# A rational definition for functional foods: A perspective

**DOI:** 10.3389/fnut.2022.957516

**Published:** 2022-09-29

**Authors:** Norman J. Temple

**Affiliations:** Centre for Science, Athabasca University, Athabasca, AB, Canada

**Keywords:** functional foods, beyond basic nutrition, novel foods, healthy foods, phytochemicals

## Abstract

Many foods are described as “functional foods”. However, the term is poorly defined. A commonly used definition is that they contain substances that have positive effects on health “beyond basic nutrition”. However, there are several problems with this definition. In many cases, healthy foods are included under the term functional foods. A new definition is proposed as follows. Functional foods are novel foods that have been formulated so that they contain substances or live microorganisms that have a possible health-enhancing or disease-preventing value, and at a concentration that is both safe and sufficiently high to achieve the intended benefit. The added ingredients may include nutrients, dietary fiber, phytochemicals, other substances, or probiotics.

## Introduction

Functional foods are poorly defined. A common definition is that they contain substances “beyond basic nutrition.” The Food and Agriculture Organization (FAO) of the United Nations uses a definition along these lines (1). They define functional foods as foods that contain, in addition to nutrients, other components that may be beneficial to health. Similarly, the Mayo Clinic defines these foods as “…. foods that have a potentially positive effect on health beyond basic nutrition” (2).

The Academy of Nutrition and Dietetics recently published a short article that discusses functional foods (3). While no specific definition is given, functional foods appear to include most healthy foods (such as fish, beans, whole grains, and nuts) as well as fortified, enriched foods. Although not explicitly stated, this apparently includes white bread. This definition is extremely broad and seems to exclude only a small minority of foods, such as sugar-sweetened beverages and alcoholic drinks.

## Problems with the definition of functional foods

We see in the above examples several problems with commonly used definitions of functional foods. The key issues are as follows:

1. The inclusion of commonly eaten healthy foods. Examples of healthy foods that have been described as functional include beetroot (4), peanuts (5), sweet potato (6), pomegranate juice (7), and strawberries (8). These foods may enhance health and prevent disease as a result of their content of phytochemicals, as well as of micronutrients. Likewise, yogurt that contains probiotics is believed to be healthy food and could be classified functional. If these foods are all classified as functional, then virtually every food recommended by food guides should also be classified as functional. For that reason, describing these healthy foods as functional appears to serve no useful purpose and merely causes confusion. It makes much more sense to refer to these foods by terms, such as “healthy foods” or “foods that may help prevent disease”.
2. Categorizing various substances as being “beyond basic nutrition” is another source of confusion. Should this term include dietary fiber? It is often unclear if the term includes nutrients. Carotenoids illustrate the problem. Carotenoids that the body can convert into retinol and therefore use as a source of vitamin A (e.g., beta-carotene) are clearly part of basic nutrition, whereas other carotenoids that have no vitamin A activity (e.g., lutein and lycopene) are arguably “beyond basic nutrition”. This makes little sense.
3. Another problem related to the concept of “beyond basic nutrition” is that with many foods believed to have health-enhancing or disease-preventing benefits, it is often unclear which components of the food are responsible for the benefit. For example, it is not known how much of health benefits linked to consumption of unrefined cereals come from nutrients, how much from fiber, and how much from phytochemicals. It is therefore misleading to refer to unrefined cereals as functional foods on the basis of their content of phytochemicals.
4. Many novel foods have been developed and marketed in recent years that contain added nutrients and are intended to enhance health. Examples include orange juice with added calcium and margarine fortified with omega-3 fatty acids. It makes good sense to classify these foods as functional, as they are distinct from conventional foods.

## Proposed new definition of functional foods

Based on the arguments presented here, a new definition is needed for functional foods. The proposed definition is as follows:

Functional foods are novel foods that have been formulated so that they contain substances or live microorganisms that have a possible health-enhancing or disease-preventing value, and at a concentration that is both safe and sufficiently high to achieve the intended benefit. The added ingredients may include nutrients, dietary fiber, phytochemicals, other substances, or probiotics.

The Institute of Food Technologists presented a similar definition (9). A key feature of these foods is that they are novel. This therefore excludes foods such as yogurt and refined cereals with added B vitamins.

## Examples of functional foods

Examples of foods that can be classed as functional are as follows:

1. As mentioned above, orange juice with added calcium and margarine (and other foods) with increased levels of omega-3 fatty acids. A body of evidence suggests that supplements of omega-3 fatty acids may be protective against cardiac diseases (10), memory impairment (11), and cognitive impairment (12).
2. Studies on the microbiome and its relationship to health have gained high interest in recent years. The focus has been on the gut microbiota. Probiotics are preparations containing potentially beneficial microorganisms in a concentrated form. This may help lead to a healthy intestine. Some evidence suggests that probiotics may improve immune function (13) and assist with weight loss (14). Depending on how they are manufactured, yogurt and kefir may contain probiotics. Other fermented foods contain fungi rather than bacteria. Examples include tempeh (from Indonesia) and miso (from Japan), both of which are made from soybeans. As the above four foods have been eaten for centuries, they not are not novel and should therefore not be considered to be functional foods. However, innovative food manufacturers could formulate novel foods that are prepared from fermented foods and which contain live good microorganisms (bacteria and possibly fungi) that are likely to be beneficial to health. Such foods would meet the proposed definition of functional foods.
3. Foods with added prebiotics are another potential type of functional food. Prebiotics are substances that can exert a favorable impact on the microbiome of the colon. In this respect, they are comparable to probiotics. Beta-glucans (from oats) (15) and fructans (16) are examples of prebiotics.
4. Some brands of margarine and other foods contain added plant sterols and stanols. These substances decrease the blood level of total cholesterol and low-density lipoprotein cholesterol (17).
5. Findings from many studies suggest that tea has a beneficial effect on health. In particular, it appears to be protective against obesity, metabolic syndrome, type 2 diabetes, cardiovascular disease, cancer, and possibly some neurodegenerative diseases (18). The class of substances believed to be responsible for this benefit is catechins. These findings suggest that tea extracts could be added to selected foods in order to manufacture functional foods. Catechins belong to a class of phenolics known as flavonoids. More generally, phenolics are phytochemicals. These are non-vitamin organic substances present in food and are believed to have beneficial effects on health.
6. Much evidence indicates that berries are beneficial for health and are protective against a variety of diseases (19–22). Anthocyanins are widely distributed in berries and are believed to be responsible for health benefits (23, 24). Anthocyanins, therefore, could potentially be utilized in the manufacture of functional foods. Like catechins, anthocyanins are also flavonoids.

The term nutraceuticals is often used for substances with claimed health-enhancing properties. The substances referred to in examples 3–6 above can be categorized as nutraceuticals.

## Research challenges

There is a need for much more high-quality evidence before we can justify the marketing of any of the examples of functional foods suggested above. Specifically, four types of evidence are needed:

1. In some of the above cases, such as catechins and omega-3 fatty acids, the active ingredient is not a clearly identified substance but a group of related substances. Similarly, various types of bacteria can be used in probiotics. Research is therefore needed to identify the most appropriate active ingredient (or combination of active ingredients).
2. In most cases, the most appropriate concentration of the active ingredient has not been established. This is clearly another area where more research is needed.
3. Research is needed in order to firmly demonstrate that functional food achieve the claimed health benefits.
4. The safety of functional foods must also be firmly demonstrated.

Randomized controlled trials are the most appropriate type of research study. This is especially the case with the third and fourth areas of research.

The crucial need for solid research, especially in the third and fourth areas of research, is exemplified by the case of dietary supplements of calcium. These are often taken by middle-aged and older women in the hope that they will help protect against osteoporosis. However, there is little supporting evidence for this (25). Moreover, their use has been linked to increased risk of cardiovascular disease in postmenopausal women (26).

## The marketing of functional foods

One potential danger of functional foods is that food companies could make false claims that a food is functional as a tool to boost sales. There are many great examples of food companies making misleading or bogus health claims when marketing foods. This is a common practice in the marketing of dietary supplements (27, 28). Vitamin water provides another example. This beverage first appeared in 2000 and comes in a variety of formulations. It contains roughly 5.5 g of sugar per 100 ml, about half the concentration found in Coca-Cola. It also contains vitamins and other assorted substances. The manufacturers have succeeded in achieving the best of each world: consumers have the taste of a sugary beverage while the product's name and ingredients imply that it is healthy. This product illustrates how functional foods could be marketed in a dishonest way.

## Data availability statement

The original contributions presented in the study are included in the article/supplementary material, further inquiries can be directed to the corresponding author.

## Author contributions

The author confirms being the sole contributor of this article and has approved it for publication.

## Conflict of interest

The author declares that the research was conducted in the absence of any commercial or financial relationships that could be construed as a potential conflict of interest.

## Publisher's note

All claims expressed in this article are solely those of the authors and do not necessarily represent those of their affiliated organizations, or those of the publisher, the editors and the reviewers. Any product that may be evaluated in this article, or claim that may be made by its manufacturer, is not guaranteed or endorsed by the publisher.
